# Health-related quality of life in patients surgically treated for orbital blow-out fracture: a prospective study

**DOI:** 10.1007/s10006-020-00923-x

**Published:** 2020-12-05

**Authors:** Hanna Rajantie, Leena Kaukola, Johanna Snäll, Risto Roine, Harri Sintonen, Hanna Thorén

**Affiliations:** 1 Department of Oral and Maxillofacial Diseases, University of Helsinki and Helsinki University Hospital, Helsinki, Finland; 2 Department of Health and Social Management, University of Eastern Finland, Kuopio, Finland; 3 Helsinki and Uusimaa Hospital District, Administration, Helsinki, Finland; 4 Department of Public Health, University of Helsinki, Helsinki, Finland; 5 Department of Oral and Maxillofacial Surgery, University of Turku, Turku, Finland; 6 Department of Oral and Maxillofacial Diseases, Turku University Hospital, Turku, Finland

**Keywords:** Facial trauma, Orbital fracture, Orbital reconstruction, Patient satisfaction, Health-related quality of life

## Abstract

**Purpose:**

The purpose of this study was to evaluate patients’ health-related quality of life (HRQoL) before and after surgical treatment of orbital blow-out fracture.

**Methods:**

This prospective study comprises of all adult patients undergoing a surgical reconstruction of an orbital blow-out fracture in 2006–2010. Their HRQoL was evaluated for 6 months postoperatively with the aid of the standardized 15D instrument and was compared with that of an age- and gender-standardized sample of the general Finnish population. A complementary questionnaire for more detailed information was also administered.

**Results:**

Twenty-six patients completed the study. Mean 15D score among the patients preoperatively (0.898) was statistically significantly and clinically importantly worse than the score of the control population (0.936). Six months postoperatively, the mean 15D score was 0.920, with no significant difference compared with the control population and the significant differences on the different dimensions had disappeared. The most common complaint at 6 months postoperatively was diplopia in daily life (19%). Disturbances in facial sensation (27%) and defects in facial appearance (15%) were the most unpleasant subjective outcomes.

**Conclusion:**

The HRQoL is significantly decreased after orbital blow-out fracture compared with the general population but will recover completely in 6 months. Thus, the negative impact of orbital blow-out fracture on HRQoL is only transient. Disturbances in facial sensation, defects in facial appearance, and diplopia are the most common subjective complaints after the injury and its surgical treatment. However, these do not appear to affect the overall quality of life in the long term.

**Supplementary Information:**

The online version contains supplementary material available at 10.1007/s10006-020-00923-x.

## Introduction

Orbital fracture is common in patients with facial trauma [1, 2]. Facial trauma can cause esthetic and functional defects as well as psychological distress and significant emotional, social, and behavioral problems, therefore, having a great impact on the quality of life of the patients [3–5]. Moreover, patients are more likely to have marital conflicts, problems with alcohol consumption, legal problems, and deficits in occupational functions after facial trauma [6].

Patients with orbital fracture confront many potential problems that may affect their quality of life, such as disturbances in vision, changes in facial appearance, sensory disturbances, impairment of the lacrimal excretion system and functions of eyelids, prolonged facial pain, anxiety, depression, and interruptions in social and professional life [7, 8]. Surgical interventions can also expose patients to different disadvantages. Quality of life is established as an important outcome for evaluating the impact of any disease and for assessing the efficacy of any treatment. Therefore, the aim of this study was to evaluate patients’ health-related quality of life (HRQoL) before and after surgical treatment of orbital blow-out fracture compared with that of the age- and gender-standardized general population.

## Materials and methods

This study is part of a larger cohort of patients surgically treated for different types of facial fractures in one trauma center. Included in the present study were all adult (age at least 18 years) patients with an isolated, orbital blow-out fracture needing surgical reconstruction at the primary stage. The patients were recruited over a 4-year period in 2006–2010 at the Department of Oral and Maxillofacial Surgery, Helsinki University Hospital, and were followed-up postoperatively for 6 months. Patients undergoing surgery for any other facial fracture or with infected fractures were excluded. During the study period, 28 patients met the inclusion criteria. Two patients refused to participate. Therefore, 26 patients were included in the final analysis.

HRQoL was evaluated with the 15D instrument [9], which is a comprehensive, standardized, self-administered measure of HRQoL that is considered conceptually consistent with the definition of health by the World Health Organization. The 15D questionnaire comprises the dimensions of mobility, vision, hearing, breathing, sleeping, eating, speech, excretion, usual activities (uact), mental function, discomfort and symptoms (disco), depression, distress, vitality, and sexual activity. Each dimension is divided into five levels that range from no problems to severe difficulties. The patients self-administered the questionnaire before the surgery (on the same day or the day before) and again on each follow-up visit at 1 week and at 1, 3, and 6 months postoperatively by ticking the appropriate box of the level best describing their current health status. These individual values were then converted into dimension level values and single index scores (15D scores) with the range 0–1, with 1 representing full health and 0 being equivalent to dead. The valuation system is based on a multi-attribute utility theory and is calculated using a set of population-based preference or utility weights [9]. The dimension level values and 15D scores of the patients were compared with those of an age- and gender-standardized sample of the general Finnish population [10]. The data for the general population were obtained from the catchment area of the Helsinki University Hospital (*n* = 1108) in the National Health 2011 Health Examination Survey, which covers a representative sample of the Finnish population aged 18 years and over. A change of difference of ± 0.015 in the 15D score is considered clinically important, being the smallest amount of change a person can detect [11]. If the patient did not attend the follow-up visit, the questionnaire was left uncompleted.

A complementary questionnaire for more detailed information on patients’ perceptions concerning their recovery and how satisfied they were with the esthetic and functional outcomes of the injury and its surgery was also administered during the follow-up visits at 1 week, and at 1, 3, and 6 months postoperatively. The questionnaire consisted of seven multiple-choice questions of the patients’ perceptions regarding facial appearance, facial sensation, occlusion, chewing, diplopia, and overall recovery. For each of these parameters, the respondents chose from the following options the one that best described their current health status: (1) poor outcome, (2) moderate outcome, and (3) satisfactory outcome and for diplopia yes or no. In the last assessment at 6 months, there were also two additional questions concerning satisfaction with treatment, where patients could choose multiple responses to questions of what the most unpleasant outcome of the injury and its treatment was.

### Statistical analyses

Data were analyzed using SPSS for Windows software version 22.0 (SPSS, Inc., Chicago, IL, USA). The results are given as means with standard deviations (SDs) and 95% confidence intervals (95% CI) for the differences in 15D scores between patients and general population, and partly as medians. The significance of the differences in means between baseline and follow-up HRQoL scores was analyzed using paired samples *t* test, the differences in means between patients and the general population with the independent samples *t* test, or differences in distributions (medians) with Mann-Whitney *U* test. *p* values less than 0.05 were considered significant.

### Ethics approval

The study protocol was approved by the Ethics Committee of Helsinki University Hospital and the Internal Review Board of the Head and Neck Center, Helsinki University Hospital (Dno 33/E6/06). Written informed consent was obtained from all participants.

## Results

Of the included 26 patients, 12 had a fracture of orbital floor and 14 had fractures of both orbital floor and medial wall combined. One patient had fractures in both orbits. All patients underwent a CT imaging pre- and postoperatively. Based on CT imaging, the maximum extension of the fracture ranged between 17 and 34 mm (mean 24.0 mm, standard deviation 5.7) in the anteroposterior axis and 12 and 37 mm (mean 20.5 mm, standard deviation 8.1) in the mediolateral axis. One patient received fracture re-reconstruction performed on the first postoperative day after primary surgery, due to suboptimal primary surgery.

Table 1 shows the characteristics of the 26 patients. The mean age of patients was 48.6 (standard deviation 14.4, range 22.5–74.1) years. A slight majority (14/26, 54%) of the patients was men. The most common cause of injury was assault (13/26, 50%).

**Table 1:** Characteristics of the 26 patients with an operated orbital fracture

Predictor	Mean (standard deviation)	Range
Age (years)	48.6 (14.4)	22.5–74.1
	Number of patients	Percentage of all patients %
Gender		
Men	14	53.8
Women	12	46.2
Cause of injury		
Assault	13	50.0
Falling	12	46.2
Traffic	1	3.8
Approach		
Infraorbital	2	7.7
Subciliar	2	7.7
Subtarsal	7	26.9
Transconjuctival	15	57.7
Reoperation	1	3.8

The response rate to the 15D questionnaire varied at different follow-up visits and between questions from 77% (20 patients) to 96% (25 patients) and to the additional questionnaire from 73% (19 patients) to 81% (21 patients).

**Fig. 1:**
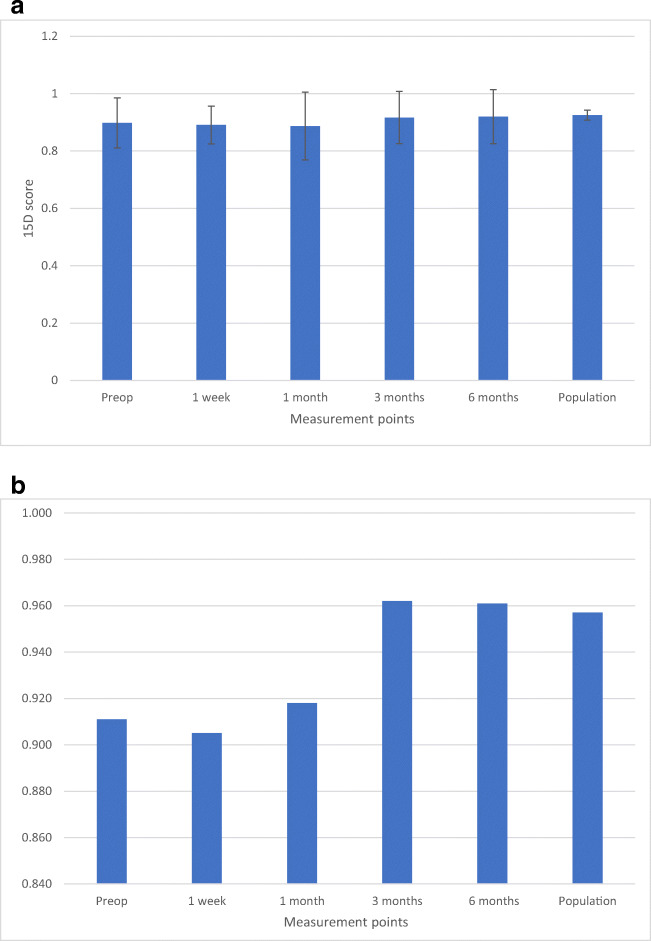
**a** The mean 15D score was preoperatively 0.898 (statistical significance of the difference from the control population *p* = 0.046), at 1 week 0.892 (*p* = 0.007), at 1 month 0.887 (0.071), at 3 months 0.921 (*p* = 0.062), and at 6 months 0.920 (0.559). The mean 15D score of the population standardized for age and gender was 0.936. **b** The median 15D score was preoperatively 0.911 (statistical significance of the difference in the distribution/median from the control population *p* = 0.171), at 1 week 0.905 (*p* = 0.027), at 1 month 0.918 (*p* = 0.362), at 3 months 0.962 (*p* = 0.183), and at 6 months 0.961 (*p* = 0.368). The median 15D score of the population standardized for age and gender was 0.957

Figure 1a shows the progression of the mean 15D scores at each follow-up visit compared with the score of the age- and gender-standardized population (mean score 0.936). The mean 15D score was preoperatively 0.898, at 1 week 0.892, at 1 month 0.887, at 3 months 0.921, and at 6 months 0.920. The mean 15D score of the patients was preoperatively (*p* = 0.046) and 1 week postoperatively (*p* = 0.007) statistically significantly and clinically importantly lower than that of the control population: the 95% CI of the difference was 0.001–0.074 preoperatively and 0.012–0.070 at 1 week.At 1, 3, and 6 months postoperatively, there was no statistically significant difference from the control population: the 95% CI of the difference was − 0.004–0.099 at 1 month, − 0.033–0.053 at 3 months, and − 0.031–0.056 at 6 months.

Figure 1b, based on median scores, confirms the same pattern: at 1, 3, and 6 months postoperatively, there was no statistically significant difference in the distribution/median from the control population.

**Fig. 2:**
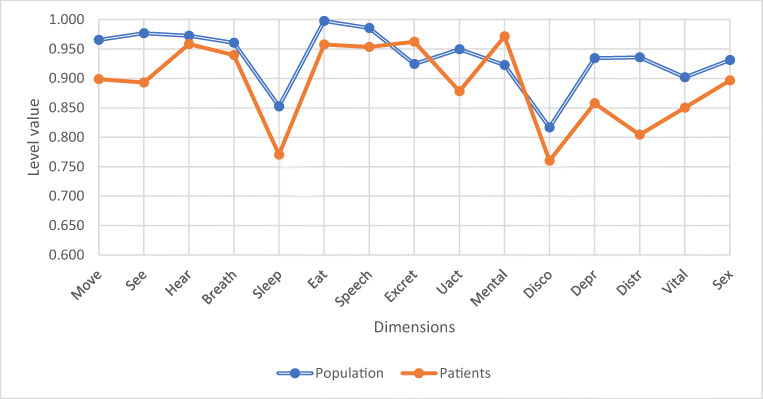
There was a significant difference on six dimensions: moving (*p* = 0.047), vision (0.005), sleeping (*p* = 0.030), mental function (*p* = 0.021), depression (*p* = 0.021), and distress (*p* = 0.001). Other dimensions did not differ significantly (hearing *p* = 0.581, breathing *p* = 0.408, eating *p* = 0.101, speech *p* = 0.252, excretion *p* = 0.93, usual activities *p* = 0.104, discomfort *p* = 0.237, vitality *p* = 0.138, sexuality *p* = 0.438)

Figure 2 shows the preoperative 15D dimensions of the patients compared with the general population. The 15D scores differed significantly on six dimensions: moving (*p* = 0.047), vision (*p* = 0.005), sleeping (*p* = 0.030), mental function (*p* = 0.021), depression (*p* = 0.021), and distress (*p* = 0.001). Of these, mental function was better among patients; on all other five dimensions, they were worse off than the general population.

**Fig. 3:**
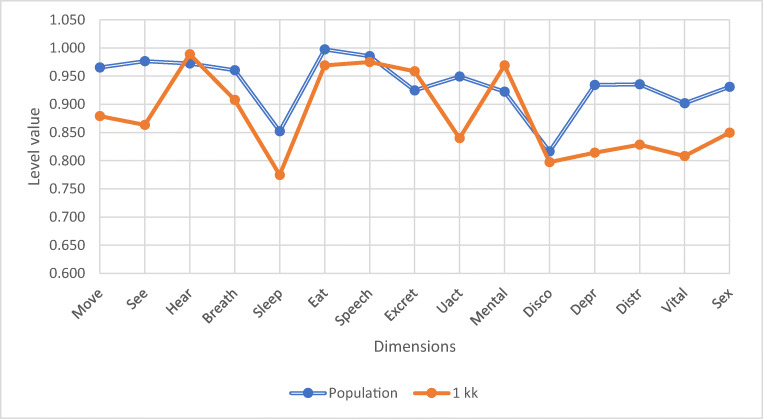
There was a significant difference on seven dimensions: moving (*p* = 0.037), vision (*p* = 0.006), usual activities (*p* = 0.012), mental function (*p* = 0.033), depression (*p* = 0.025), distress (*p* = 0.032) and vitality (*p* = 0.047). Other dimensions did not differ significantly (hearing *p* = 0.169, breathing *p* = 0.103, sleeping *p* = 0.126, eating *p* = 0.196, speech *p* = 0.699, excretion *p* = 0.141, discomfort *p* = 0.635, sexuality *p* = 0.164)

One month postoperatively, patients’ dimension scores differed significantly from those of the controls on 7 dimensions: moving (*p* = 0.037), vision (*p* = 0.006), usual activities (*p* = 0.012), mental function (*p* = 0.033), depression (*p* = 0.025), distress (*p* = 0.032), and vitality (*p* = 0.047), as shown in Fig. 3. Again, mental function was better among patients, and on the rest of these dimensions, they were worse off than the control population.

**Fig. 4:**
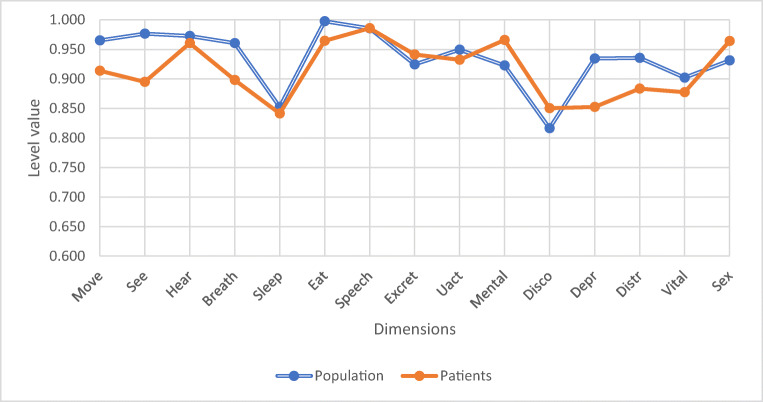
The only dimension with a significant difference was vision (*p* = 0.045). Other dimensions did not differ significantly (moving *p* = 0.161, hearing *p* = 0.775, breathing *p* = 0.158, sleeping *p* = 0.901, eating *p* = 0.195, speech *p* = 0.990, excretion *p* = 0.479, usual activities *p* = 0.721, mental function *p* = 0.06, discomfort *p* = 0.392, depression *p* = 0.053, distress *p* = 0.165, vitality *p* = 0.585, sexuality *p* = 0.311)

Three months postoperatively, the only dimension with a significant difference compared with the general population was vision (*p* = 0.045), as presented in Fig. 4.

**Fig. 5:**
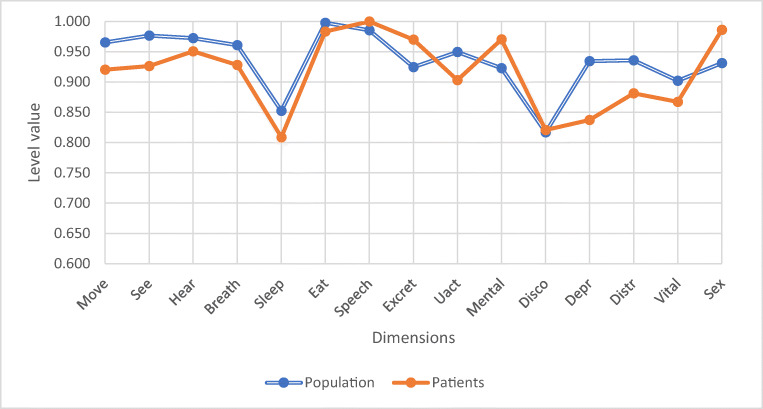
None of the dimension scores was significantly lower. The dimensions with a significantly higher mean score were excretion (*p* = 0.030) and sexual activity (*p* = 0.001). Other dimensions did not differ significantly (moving *p* = 0.250, vision *p* = 0.182, hearing *p* = 0.496, breathing *p* = 0.373, sleeping *p* = 0.375, eating *p* = 0.403, speech *p* = 0.00, usual activities *p* = 0.360, mental function *p* = 0.107, discomfort *p* = 0.880, depression *p* = 0.074, distress *p* = 0.297, vitality *p* = 0.462)

Six months postoperatively, none of the dimension scores was significantly lower in patients than in the general population. Dimensions with significantly higher mean scores were excretion (*p* = 0.030) and sexual activity (*p* = 0.001), as shown in Fig. 5. (The means and standard deviations of the dimension scores at different measurement points are shown in a supplementary file).

**Fig. 6:**
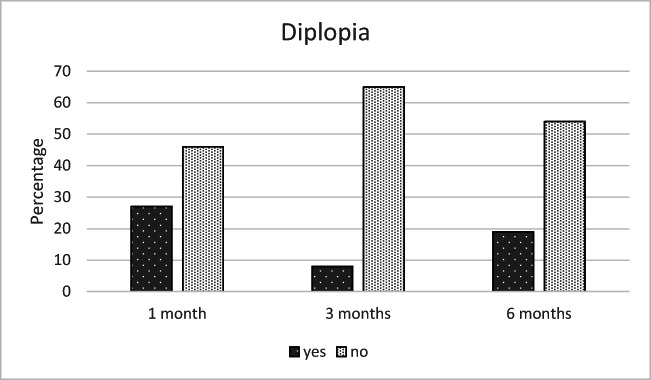
The most unpleasant outcome from the injury reported by the patients (% of the 26 patients)

**Fig. 7:**
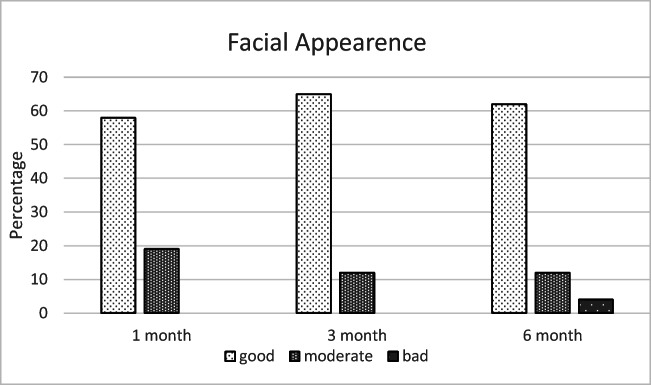
Patients’ assessment on facial appearance at different time points (% of the 26 patients)

**Fig. 8:**
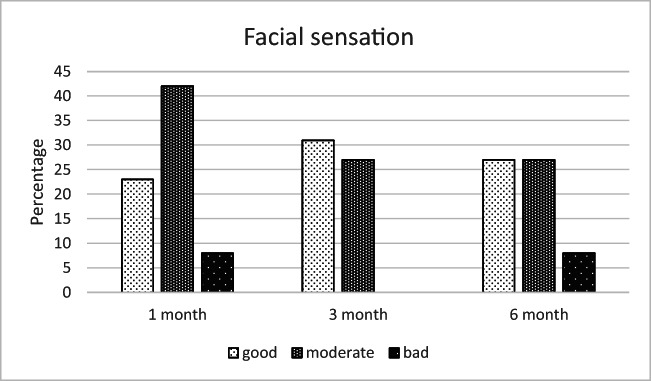
Patients’ assessment on facial sensation at different time points (% of the 26 patients)

**Fig. 9:**
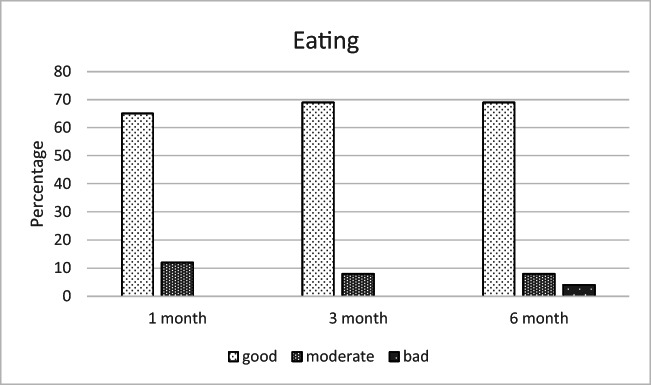
Patients’ assessment on eating at different time points (% of the 26 patients)

According to the responses to the complementary questionnaire, the most common complaints at 6 months postoperatively were diplopia (19%), disturbances in facial sensation (8%), defects in facial appearance (4%), and difficulties in eating (4%), as shown in Figs. 6, 7, 8, and 9.

All of the five patients (19%), who reported diplopia 6 months after trauma, experienced mild or moderate diplopia occasionally in daily life. Diplopia could not be demonstrated objectively at the follow-up visit by the clinician. One patient was nevertheless referred to an ophthalmologist, but any further procedures were not performed after more specific examinations.

**Fig. 10:**
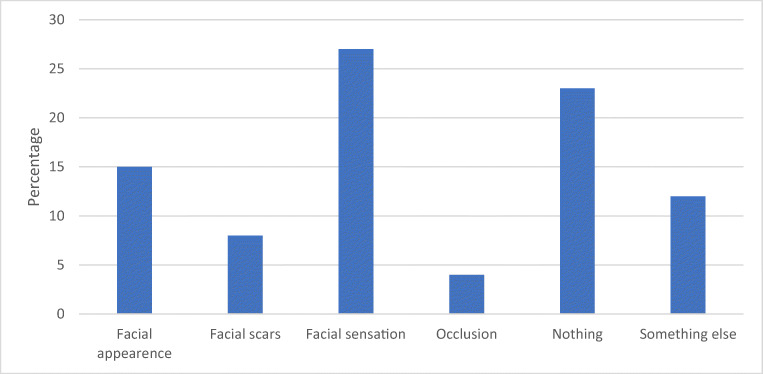
Defects in facial sensation (27%) and disturbances in facial appearance (15%) were the most unpleasant outcomes of the injury. Three patients (12%) answered something else, which were uncertainty, swelling, and defects in field of view

Moreover, patients reported especially defects in facial sensation (27%) and disturbances in facial appearance (15%) to be the most unpleasant outcome of the injury (see Fig. 10). However, 23% of the respondents also reported no complications at all and were fully satisfied with the treatment and the outcome. One patient (4%) reported problems in occlusion. This patient had been assaulted and was diagnosed with temporomandibular dysfunction (TMD) during the follow-up visits.

The most unpleasant symptom caused by the treatment itself was considered by the patients to be swelling (46%), pain (27%), or stress (27%).

## Discussion

The primary objective of the study was to evaluate prospectively, with the 15D instrument, HRQoL before and after surgical reconstruction of orbital blow-out fracture during a 6-month follow-up. A further objective was to document, with the aid of a specifically designed questionnaire, patients’ perceptions of esthetic and functional outcomes after the injury and its treatment.

The prevalence of maxillofacial trauma is known to be higher in men than in women [1]. However, previous studies have demonstrated that in orbital fracture patients’ gender distribution is more equal than in other types of facial fractures [12]. This was also the case in our study. The male-to-female ratio in this study was 1.17:1.

In addition, earlier studies have shown that the mean age of trauma patients is increasing, especially in developed countries [12]. This is considered to be due to the longer life expectancy of people worldwide and the resulting increase in the number of older people in the general population. Moreover, orbital fractures are known to be more frequent and severe in elderly patients, female patients predominating in this group [2, 13]. In our study, the mean age of patients was 48.7 years, which exceeds the mean age (29.9–43.9 years) of European patients with maxillofacial fractures [1]. The age of the patients in this study ranged from 22.5 to 74.1 years, reflecting the variation in etiologies of the injury. In assaults, younger patients and men are known to dominate, whereas falls are more common in older and female patients [1, 2].

The distribution of the etiologies of the trauma resembled that found in previous studies, with assault being the most common cause of injury, followed by falls and traffic accidents. The most common approach was transconjuctival (57.7%), and it was chosen by the surgeon based on the patient, location, and extent of the fracture. It needs to be noted that the fractures were thus not identical, and the technique was not standardized.

Our results revealed that HRQoL decreases significantly after orbital blow-out fracture. The mean 15D score of patients before surgery was significantly lower than that of the age- and gender-standardized population. Patients were significantly worse off on five dimensions. Our findings are in line with similar studies by Kaukola et al. [14, 15] of HRQoL after mandibular and zygomatic fractures measured with the aid of the 15D instrument. After mandibular fracture, the patients were worse off on nine dimensions and after zygomatic fracture on six dimensions compared with the general population. The decline in the mean 15D score compared with the control population after mandibular fracture (0.073) was larger than the decline after zygomatic fracture (0.021). In this study, the decline in the mean 15D score after orbital fracture (0.038) was intermediate relative to mandibular and zygomatic fractures. Thus, HRQoL decreases after an orbital blow-out fracture slightly less than after a mandibular fracture, but more than after a zygomatic fracture.

Sharma et al. [8] assessed vision-related quality of life after orbito-facial trauma in 100 patients in India with the NEI VFQ-25 questionnaire and found that 6 months after the trauma the majority of patients experienced a significant decrease in quality of life. Of the patients, 84% reported scores under 50, and of these 49% reported scores under 25, reflecting the strong decreasing impact of the trauma on the quality of life of the patients. Disturbances in sleep pattern, social interaction, and workplace functions were noted in these patients. General health was experienced as poor, and difficulties in daily activities and vision problems correlated with the trauma. Of the patients, 82% reported being frustrated since the trauma.

Ukpong et al. [5] also reported long-term negative changes in HRQoL after maxillofacial trauma.

In contrast to Sharma et al. [8] and Ukpong et al. [5], the HRQoL of our patients recovered completely in 6 months after surgical treatment. The significance of the difference between the patients and the general population disappeared quickly, after 1 month. Six months postoperatively, the mean 15D score of the patients did not differ significantly from that of the control population, and none of the dimension scores was significantly lower than those in controls. Thus, based on our findings, the negative impact of an orbital trauma on patients’ HRQoL was only temporary.

Sikora et al. [16] observed similarly a negative impact of maxillofacial trauma on the quality of life, followed by the tendency to recover with treatment. The factors affecting HRQoL have been analyzed, with the aid of the SF-36 questionnaire, immediately and 3 months after the treatment of maxillofacial fractures in 227 patients. The results showed that 3 months after treatment all domains of the HRQoL had improved significantly compared with the first evaluation. Furthermore, Sikora et al. proposed that age and gender of the patients and location and type of fracture may be important factors affecting HRQoL after maxillofacial trauma. Men and younger patients rated their quality of life as higher, but when considering the improvement during the study period, no significant difference was present between men and women. There was a significant positive correlation between older age and general health domain regarding improvement in quality of life. Comminuted and mandibular fractures were associated with a greater improvement in the quality of life during the study period.

As mentioned previously, preoperatively the patients in this study were significantly worse off on five dimensions of the 15D instrument: moving, vision, sleeping, depression, and distress. One month postoperatively, also usual activities and vitality scores were significantly affected. Surprisingly, preoperatively and after 1 month, the mental function score was higher in patients than in controls. It could be hypothesized that this result could reflect the proneness to substance abuse among patients with maxillofacial trauma [17]. When these patients are hospitalized and treated by a multidisciplinary team, they are restricted from detrimental social contacts and shielded from intoxicants and harmful behavior, possibly leading to detoxication and better mental functioning than they may experience during normal life conditions. None of the patients in the present study reported daily alcohol and/or drug consumption; however, further consumption evaluation or a mini intervention was not conducted.

Six months after the trauma, excretion and sexual activity also yielded significantly higher values than those of the general population. This effect could be due to recovery from psychological distress and physical trauma leading to a better appreciation of health.

At the end of treatment, a defect in facial sensation was mentioned by 27% of the patients as the most significant subjective problem. It needs to be noted that sensory recovery is still possible after 6 months due to nerve regeneration [18]. Although sensory disturbances are not considered as disabling as esthetic or functional defects, they are common complaints after maxillofacial trauma and patients need to be informed about them at an early stage to help them adapt to a potentially permanent disturbance. The second most common subjective complaint was reported by 15% of patients to be disturbances in facial appearance. Minor changes in globe position and eyelids may be noticeable by the patient, but more severe eyelid scarring is also possible after orbital surgery [19]. In the present study, one patient received additional surgery for entropion.

Mild or moderate diplopia in daily life was reported by 19% of the patients 6 months after the trauma. Alhamadani et al. [20] studied retrospectively diplopia and ocular motility in patients with orbital blow-out fracture during a 10-year study period and found diplopia to be very common, with a prevalence of 80%, after surgical treatment. Moreover, they concluded that surgery alone did not provide an ideal solution for diplopia as it could not address the real cause of diplopia, which was thought to be disruption of the ligament system and septa. Therefore, the authors recommended that surgical intervention should not be based on diplopia alone. Postoperative diplopia is thus a possible, unfortunate postoperative discomfort, with only minor impact on patients’ quality of life.

One patient reported problems in occlusion 6 months postoperatively. This patient had been assaulted and was diagnosed with temporomandibular dysfunction (TMD) during the follow-up visits. According to previous studies, TMD is common after mandibular and zygomatic trauma [21, 22]. In this case, it could be hypothesized that during the assault this patient received multiple forces towards the face, and some could have been directed to or transmitted to the temporomandibular joint (TMJ), causing soft tissue damage to the area without fracture of bones. Interestingly, TMD problems can therefore be associated with orbital injury as well and should be evaluated when treating any maxillofacial trauma.

The findings of this study and previously published papers [5, 8, 16] indicate that psychological and social problems are frequent among patients recovering from facial trauma. It is important to emphasize multiprofessional collaboration in providing services that support patient recovery. At our hospital, trauma patients with obvious socioeconomic challenges are routinely referred to the hospital’s social worker for further assistance.

## Conclusion

HRQoL is significantly decreased after orbital blow-out fracture compared with that of the general population but will recover completely in 6 months. Thus, the negative impact of orbital blow-out fracture on HRQoL is only transient. Evaluation of patients’ mental status and well-being is important during the early follow-up visits after the injury and surgery. Orbital blow-out fracture is a severe injury with possible long-term disadvantages. Disturbances in facial sensation, defects in facial appearance, and diplopia in daily life are the most common subjective complaints after injury and its surgical treatment. However, these do not appear to affect overall HRQoL in the long term. It has to be emphasized though that due to heterogeneity of the cohort and the rather small sample size, the results should be considered as a first step in research in this direction on HRQoL in trauma patients.

## Supplementary Information

ESM 1 (DOCX 35 kb)

## Data Availability

Not applicable.
