# Supplementary material for: Health-related quality of life in patients surgically treated for orbital blow-out fracture: a prospective study
Source: Oral Maxillofac Surg. 2020 Dec 5;25(3):373–82. doi: 10.1007/s10006-020-00923-x (PMC8352817; doi:10.1007/s10006-020-00923-x)
Supplement: Supplementary file 1 — (DOCX 35 kb) [file 10006_2020_923_MOESM1_ESM.docx]

**The means and standard deviations of the dimension level values and the 15D score of the groups (group 0=population, group 1=patients) at baseline**

|  | group | Mean | Std. Deviation | Std. Error Mean |
| --- | --- | --- | --- | --- |
| MOVE | .00 | .96529 | .026383 | .005277 |
|  | 1.00 | .89853 | .157772 | .031554 |
| SEE | .00 | .97660 | .013373 | .002675 |
|  | 1.00 | .89320 | .135619 | .027124 |
| HEAR | .00 | .97247 | .021052 | .004210 |
|  | 1.00 | .95842 | .124581 | .024916 |
| BREATH | .00 | .96068 | .023859 | .004772 |
|  | 1.00 | .93952 | .123454 | .024691 |
| SLEEP | .00 | .85248 | .033938 | .006788 |
|  | 1.00 | .77045 | .175243 | .035049 |
| EAT | .00 | .99763 | .002378 | .000476 |
|  | 1.00 | .95754 | .117342 | .023468 |
| SPEECH | .00 | .98557 | .009766 | .001953 |
|  | 1.00 | .95355 | .136095 | .027219 |
| EXCRET | .00 | .92462 | .031651 | .006330 |
|  | 1.00 | .96214 | .104640 | .020928 |
| UACT | .00 | .94948 | .023973 | .004795 |
|  | 1.00 | .87830 | .209384 | .041877 |
| MENTAL | .00 | .92259 | .025878 | .005176 |
|  | 1.00 | .97147 | .098738 | .019748 |
| DISCO | .00 | .81665 | .038491 | .007698 |
|  | 1.00 | .76016 | .230186 | .046037 |
| DEPR | .00 | .93447 | .019000 | .003800 |
|  | 1.00 | .85783 | .154444 | .030889 |
| DISTR | .00 | .93573 | .017982 | .003596 |
|  | 1.00 | .80434 | .163893 | .032779 |
| VITAL | .00 | .90196 | .021959 | .004392 |
|  | 1.00 | .85034 | .166859 | .033372 |
| SEX | .00 | .93120 | .033406 | .006681 |
|  | 1.00 | .89649 | .217407 | .043481 |
| D15SCORE | .00 | .93558 | .017476 | .003495 |
|  | 1.00 | .89825 | .087484 | .017497 |

**The means and standard deviations of the dimension level values and the 15D score of the groups (group 0=population, group 1=patients) at 1 week**

|  | group | Mean | Std. Deviation | Std. Error Mean |
| --- | --- | --- | --- | --- |
| MOVE | .00 | .96069 | .030725 | .006272 |
|  | 1.00 | .91019 | .187056 | .038183 |
| SEE | .00 | .97549 | .013421 | .002740 |
|  | 1.00 | .83151 | .146233 | .029850 |
| HEAR | .00 | .96985 | .023063 | .004708 |
|  | 1.00 | .95669 | .126953 | .025914 |
| BREATH | .00 | .95581 | .028584 | .005835 |
|  | 1.00 | .93700 | .125451 | .025608 |
| SLEEP | .00 | .85038 | .034969 | .007138 |
|  | 1.00 | .85120 | .182151 | .037181 |
| EAT | .00 | .99746 | .002378 | .000485 |
|  | 1.00 | .97052 | .099888 | .020390 |
| SPEECH | .00 | .98579 | .009216 | .001881 |
|  | 1.00 | 1.00000 | .000000 | .000000 |
| EXCRET | .00 | .91985 | .035082 | .007161 |
|  | 1.00 | .94742 | .120109 | .024517 |
| UACT | .00 | .94622 | .026436 | .005396 |
|  | 1.00 | .80118 | .156472 | .031940 |
| MENTAL | .00 | .91867 | .029399 | .006001 |
|  | 1.00 | .97028 | .100679 | .020551 |
| DISCO | .00 | .81233 | .039172 | .007996 |
|  | 1.00 | .72647 | .151532 | .030931 |
| DEPR | .00 | .93425 | .019475 | .003975 |
|  | 1.00 | .86233 | .138737 | .028320 |
| DISTR | .00 | .93572 | .018239 | .003723 |
|  | 1.00 | .85228 | .158720 | .032398 |
| VITAL | .00 | .89852 | .021854 | .004461 |
|  | 1.00 | .79760 | .127873 | .026102 |
| SEX | .00 | .92707 | .033518 | .006842 |
|  | 1.00 | .82150 | .249188 | .050865 |
| D15SCORE | .00 | .93276 | .019634 | .004008 |
|  | 1.00 | .89161 | .066345 | .013543 |

**The means and standard deviations of the dimension level values and the 15D score of the groups (group 0=population, group 1=patients) at 1 month**

|  | group | Mean | Std. Deviation | Std. Error Mean |
| --- | --- | --- | --- | --- |
| MOVE | .00 | .96348 | .031320 | .006531 |
|  | 1.00 | .87927 | .180275 | .037590 |
| SEE | .00 | .97730 | .013726 | .002862 |
|  | 1.00 | .86347 | .181180 | .037779 |
| HEAR | .00 | .97260 | .021986 | .004584 |
|  | 1.00 | .98912 | .052191 | .010883 |
| BREATH | .00 | .95837 | .029490 | .006149 |
|  | 1.00 | .90797 | .142271 | .029665 |
| SLEEP | .00 | .85288 | .034120 | .007115 |
|  | 1.00 | .77453 | .234458 | .048888 |
| EAT | .00 | .99762 | .002475 | .000516 |
|  | 1.00 | .96923 | .101931 | .021254 |
| SPEECH | .00 | .98497 | .010433 | .002175 |
|  | 1.00 | .97531 | .118394 | .024687 |
| EXCRET | .00 | .92323 | .034634 | .007222 |
|  | 1.00 | .95885 | .108642 | .022654 |
| UACT | .00 | .94883 | .026392 | .005503 |
|  | 1.00 | .83981 | .190549 | .039732 |
| MENTAL | .00 | .91991 | .028735 | .005992 |
|  | 1.00 | .96899 | .102738 | .021422 |
| DISCO | .00 | .81726 | .038964 | .008125 |
|  | 1.00 | .79737 | .194677 | .040593 |
| DEPR | .00 | .93344 | .018965 | .003955 |
|  | 1.00 | .81440 | .236833 | .049383 |
| DISTR | .00 | .93459 | .017765 | .003704 |
|  | 1.00 | .82837 | .222391 | .046372 |
| VITAL | .00 | .90036 | .023406 | .004880 |
|  | 1.00 | .80830 | .208591 | .043494 |
| SEX | .00 | .93219 | .031659 | .006601 |
|  | 1.00 | .84974 | .272757 | .056874 |
| D15SCORE | .00 | .93465 | .019808 | .004130 |
|  | 1.00 | .88731 | .118414 | .024691 |

**The means and standard deviations of the dimension level values and the 15D score of the groups (group 0=population, group 1=patients) at 3 months**

|  | group | Mean | Std. Deviation | Std. Error Mean |
| --- | --- | --- | --- | --- |
| MOVE | .00 | .95891 | .031656 | .006908 |
|  | 1.00 | .91387 | .134984 | .030183 |
| SEE | .00 | .97554 | .012643 | .002759 |
|  | 1.00 | .89501 | .167461 | .037445 |
| HEAR | .00 | .96886 | .023337 | .005093 |
|  | 1.00 | .96054 | .130166 | .029106 |
| BREATH | .00 | .95350 | .029665 | .006473 |
|  | 1.00 | .89826 | .166008 | .037120 |
| SLEEP | .00 | .84654 | .033305 | .007268 |
|  | 1.00 | .84130 | .188246 | .041079 |
| EAT | .00 | .99731 | .002467 | .000538 |
|  | 1.00 | .96462 | .108897 | .024350 |
| SPEECH | .00 | .98606 | .009832 | .002146 |
|  | 1.00 | .98587 | .064745 | .014129 |
| EXCRET | .00 | .91618 | .034481 | .007524 |
|  | 1.00 | .94118 | .156762 | .034208 |
| UACT | .00 | .94463 | .024919 | .005438 |
|  | 1.00 | .93220 | .155116 | .033849 |
| MENTAL | .00 | .91795 | .030895 | .006742 |
|  | 1.00 | .96604 | .107263 | .023407 |
| DISCO | .00 | .80933 | .035409 | .007727 |
|  | 1.00 | .85032 | .206849 | .046253 |
| DEPR | .00 | .93280 | .018520 | .004041 |
|  | 1.00 | .85239 | .178173 | .038881 |
| DISTR | .00 | .93426 | .017277 | .003770 |
|  | 1.00 | .88354 | .160626 | .035052 |
| VITAL | .00 | .89732 | .022142 | .004832 |
|  | 1.00 | .87760 | .161485 | .035239 |
| SEX | .00 | .92674 | .033348 | .007277 |
|  | 1.00 | .96422 | .163969 | .035781 |
| D15SCORE | .00 | .93120 | .019166 | .004182 |
|  | 1.00 | .92096 | .091005 | .020349 |

**The means and standard deviations of the dimension level values and the 15D score of the groups (group 0=population, group 1=patients) at 6 months**

|  | group | Mean | Std. Deviation | Std. Error Mean |
| --- | --- | --- | --- | --- |
| MOVE | .00 | .96072 | .031697 | .006917 |
|  | 1.00 | .92021 | .153925 | .033589 |
| SEE | .00 | .97600 | .013110 | .002861 |
|  | 1.00 | .92618 | .164800 | .035962 |
| HEAR | .00 | .97106 | .023594 | .005149 |
|  | 1.00 | .95050 | .134955 | .029450 |
| BREATH | .00 | .95486 | .029975 | .006541 |
|  | 1.00 | .92800 | .131978 | .028800 |
| SLEEP | .00 | .84953 | .037209 | .008120 |
|  | 1.00 | .80855 | .203933 | .044502 |
| EAT | .00 | .99739 | .002430 | .000530 |
|  | 1.00 | .98315 | .077205 | .016848 |
| SPEECH | .00 | .98635 | .009280 | .002025 |
|  | 1.00 | 1.00000 | .000000 | .000000 |
| EXCRET | .00 | .91986 | .036621 | .007991 |
|  | 1.00 | .96995 | .094900 | .020709 |
| UACT | .00 | .94816 | .026903 | .005871 |
|  | 1.00 | .90298 | .219413 | .047880 |
| MENTAL | .00 | .91993 | .030727 | .006705 |
|  | 1.00 | .97024 | .136386 | .029762 |
| DISCO | .00 | .81362 | .040613 | .008862 |
|  | 1.00 | .82059 | .204508 | .044627 |
| DEPR | .00 | .93292 | .019635 | .004285 |
|  | 1.00 | .83732 | .232153 | .050660 |
| DISTR | .00 | .93470 | .018387 | .004012 |
|  | 1.00 | .88117 | .228291 | .049817 |
| VITAL | .00 | .89858 | .023113 | .005044 |
|  | 1.00 | .86714 | .190594 | .041591 |
| SEX | .00 | .93018 | .031787 | .006936 |
|  | 1.00 | .98617 | .063392 | .013833 |
| D15SCORE | .00 | .93310 | .020474 | .004468 |
|  | 1.00 | .92055 | .094725 | .020671 |
